# Regulation of Life Cycle Checkpoints and Developmental Activation of Infective Larvae in *Strongyloides stercoralis* by Dafachronic Acid

**DOI:** 10.1371/journal.ppat.1005358

**Published:** 2016-01-04

**Authors:** Mennatallah M. Y. Albarqi, Jonathan D. Stoltzfus, Adeiye A. Pilgrim, Thomas J. Nolan, Zhu Wang, Steven A. Kliewer, David J. Mangelsdorf, James B. Lok

**Affiliations:** 1 Department of Pathobiology, School of Veterinary Medicine, University of Pennsylvania, Philadelphia, Pennsylvania, United States of America; 2 Department of Biology, Hollins University, Roanoke, Virginia, United States of America; 3 Department of Pharmacology, University of Texas Southwest Medical Center, Dallas, Texas, United States of America; 4 Department of Molecular Biology, University of Texas Southwest Medical Center, Dallas, Texas, United States of America; 5 Howard Hughes Medical Institute, University of Texas Southwest Medical Center, Dallas, Texas, United States of America; McGill University, CANADA

## Abstract

The complex life cycle of the parasitic nematode *Strongyloides stercoralis* leads to either developmental arrest of infectious third-stage larvae (iL3) or growth to reproductive adults. In the free-living nematode *Caenorhabditis elegans*, analogous determination between dauer arrest and reproductive growth is governed by dafachronic acids (DAs), a class of steroid hormones that are ligands for the nuclear hormone receptor DAF-12. Biosynthesis of DAs requires the cytochrome P450 (CYP) DAF-9. We tested the hypothesis that DAs also regulate *S*. *stercoralis* development via DAF-12 signaling at three points. First, we found that 1 μM Δ7-DA stimulated 100% of post-parasitic first-stage larvae (L1s) to develop to free-living adults instead of iL3 at 37°C, while 69.4±12.0% (SD) of post-parasitic L1s developed to iL3 in controls. Second, we found that 1 μM Δ7-DA prevented post-free-living iL3 arrest and stimulated 85.2±16.9% of larvae to develop to free-living rhabditiform third- and fourth-stages, compared to 0% in the control. This induction required 24–48 hours of Δ7-DA exposure. Third, we found that the CYP inhibitor ketoconazole prevented iL3 feeding in host-like conditions, with only 5.6±2.9% of iL3 feeding in 40 μM ketoconazole, compared to 98.8±0.4% in the positive control. This inhibition was partially rescued by Δ7-DA, with 71.2±16.4% of iL3 feeding in 400 nM Δ7-DA and 35 μM ketoconazole, providing the first evidence of endogenous DA production in *S*. *stercoralis*. We then characterized the 26 CYP-encoding genes in *S*. *stercoralis* and identified a homolog with sequence and developmental regulation similar to DAF-9. Overall, these data demonstrate that DAF-12 signaling regulates *S*. *stercoralis* development, showing that in the post-parasitic generation, loss of DAF-12 signaling favors iL3 arrest, while increased DAF-12 signaling favors reproductive development; that in the post-free-living generation, absence of DAF-12 signaling is crucial for iL3 arrest; and that endogenous DA production regulates iL3 activation.

## Author Summary

*Strongyloides stercoralis* is a parasitic nematode that infects hundreds of millions of people worldwide. The infectious form of *S*. *stercoralis* is a developmentally arrested third-stage larva (iL3); once inside the host, the iL3 activates and develops into an adult parasitic female. First-stage larvae (L1) excreted in the host feces have two routes of development: either directly to iL3 or indirectly to free-living adults. The molecular mechanisms controlling iL3 developmental arrest and activation, and the switch regulating post-parasitic L1 development, are poorly understood. The free-living nematode *Caenorhabditis elegans* has a developmentally arrested stage, morphologically similar to iL3, called dauer. Dauer formation is prevented by endogenous production of a class of steroid hormones called dafachronic acids (DAs), which are synthesized by a cytochrome P450. We demonstrated that in *S*. *stercoralis*, administering DA can both stimulate post-parasitic L1 to develop to free-living adults instead of iL3 as well as prevent iL3 developmental arrest. Additionally, blocking cytochrome P450 function prevents iL3 activation in a host-like environment, suggesting endogenous DA production. We also characterized the developmental expression of cytochrome P450s present in the *S*. *stercoralis* genome. Together, our data demonstrate that DA regulates *S*. *stercoralis* iL3 arrest and activation and the post-parasitic developmental switch.

## Introduction

*Strongyloides stercoralis* is a parasitic nematode that infects both humans and dogs and is the causative agent of strongyloidiasis, which predominately afflicts socio-economically disadvantaged people in developing countries [1–3]. While chronic strongyloidiasis is often asymptomatic or accompanied by low-grade gastrointestinal symptoms, *S*. *stercoralis* infection in immunocompromised or corticosteroid-treated patients can progress to hyperinfection and disseminated strongyloidiasis, which can be fatal [4,5]. Understanding the mechanisms regulating the development of *S*. *stercoralis* may lead to improved diagnostic, control, and treatment strategies.

**Fig 1:**
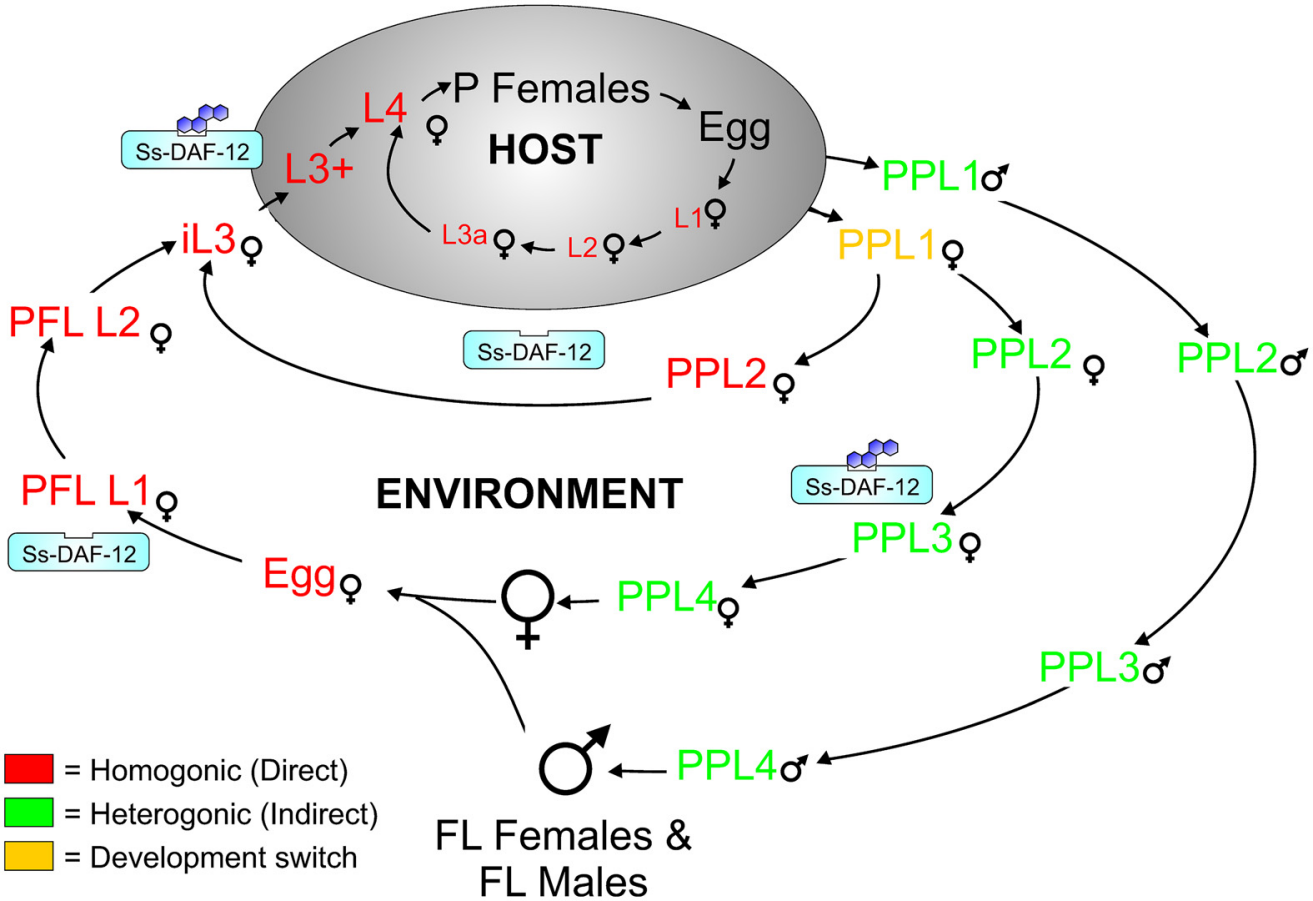
Hypothesized regulation of the *Strongyloides stercoralis* life cycle by the nuclear hormone receptor DAF-12. The *S*. *stercoralis* parasitic female (P Female) produces larval progeny by mitotic parthenogenesis, and these progeny have several possible developmental fates. A female post-parasitic first-stage larva (PP L1) can either precociously develop inside the host to an autoinfective third-stage larva (L3a), which develops to a second-generation parasitic female, or be passed in the feces to develop outside the host by one of two routes: a homogonic route directly to a developmentally arrested infectious third-stage larva (iL3), which is favored at host-like temperatures (e.g., 37°C), or a heterogonic route to a free-living adult female (FL Female), which is favored at lower temperatures (e.g., 22°C). We hypothesize that this developmental checkpoint is regulated by dafachronic acid ligands for the nuclear hormone receptor *Ss*-DAF-12, with liganded *Ss*-DAF-12 favoring heterogonic development. Larval progeny of the single free-living generation of females and males invariably form iL3, and this developmental arrest is hypothesized to be governed by the absence of *Ss*-DAF-12 signaling. Once inside a host, the third-stage larva resumes development and feeding, resulting in a form designated the L3+. We hypothesize that resumption of development by iL3 entering the host and maturation to the P Female requires an increase in signaling by *Ss*-DAF-12, stimulated by increased biosynthesis of its steroid ligand.

Similar to many nematodes, including the free-living nematode *Caenorhabditis elegans*, *S*. *stercoralis* has crucial points in its life cycle, where the organism is either fated towards reproductive adulthood or developmental arrest (Fig 1). Female post-parasitic first-stage larvae excreted in the feces of an infected host can undertake two possible routes of development: a homogonic route leading directly to developmentally arrested infectious third-stage larvae (iL3) or a heterogonic route leading to free-living adults [6]. This developmental switch is analogous to the switch between dauer arrest and reproductive development made by first-stage *C*. *elegans* larvae [7], with dauer arrest favored at high temperatures, low food abundance, and high population density, which is signaled by rising titers of constitutively-produced ascaroside pheromones [7–9]. *S*. *stercoralis* also exercises strict developmental controls in the post-free-living generation, where larvae invariably mature into non-feeding iL3; however, upon entering a permissive host, third-stage larvae resume feeding and development, eventually maturing into parthenogenetic parasitic females in the intestinal lumen [3,10,11]. Similarly, *C*. *elegans* dauer larvae resume feeding and develop to reproductive adults when environmental conditions improve [7]. Parallels between *S*. *stercoralis* iL3 and *C*. *elegans* dauer larvae extend beyond these functional similarities and include shared morphological features, including a long, radially constricted filariform pharynx, a plugged buccal cavity, and a stress-resistant cuticle [12–14]. While pathways regulating dauer arrest, and to a lesser extent dauer exit, have been well-studied in *C*. *elegans* [15], the developmental controls regulating *S*. *stercoralis* iL3 formation and activation have only recently been examined [16–20]. The "dauer hypothesis" predicts that similar mechanisms govern iL3 and dauer development [14,21]; however, given that *C*. *elegans* and *S*. *stercoralis* are members of two different nematode clades [22], with parasitism thought to have arisen independently in each [23], it is entirely plausible that different signaling mechanisms could regulate formation of *S*. *stercoralis* iL3 and *C*. *elegans* dauer larvae [14,24].

In *C*. *elegans*, one of the primary mechanisms regulating the determination between reproductive development and dauer arrest involves a class of endogenous steroid hormones known as dafachronic acids (DAs) [25]. Under conditions promoting reproductive growth and development, DAs are abundant and bind the nuclear hormone receptor *Ce*-DAF-12, which controls a network of genes that carry out these functions. Conversely, conditions favoring dauer arrest lead to a paucity of DAs and to *Ce*-DAF-12 functioning as a co-repressor, thereby instituting a genetic program for developmental arrest [26]. This mutually exclusive developmental switch occurs in the first-stage larvae (L1) and must be reinforced to prevent development of worms with both dauer and adult attributes, which would be detrimental to the organism. When an L1 encounters favorable conditions, environmental cues—transduced by upstream cyclic guanosine monophosphate (cGMP) signaling, and then by parallel insulin/insulin-like growth factor (IIS) and dauer transforming growth factor β (TGFβ) signaling pathways [8,15]—trigger DA production in neuroendocrine XXX cells in the head of the developing larva [27]. The initial small quantity of DAs produced by XXX cells promotes further DA production throughout the hypodermis via a *Ce*-DAF-12-mediated positive feedback loop of DA synthesis, thereby ensuring the worm commits to reproductive development [28–30]. In *C*. *elegans*, the key enzyme in endogenous DA biosynthesis is the cytochrome P450 *Ce*-DAF-9 [28,31,32], which is reflected in a significant increase in *Ce-daf-9* transcripts during reproductive development and dauer exit [29,33].

Similar to steroids in other animals, *C*. *elegans* DAs are derived from cholesterol [25], which is first modified by the Rieske-like oxygenase *Ce*-DAF-36 [34], and subsequently by the 3-hydroxysteroid dehydrogenase *Ce*-DHS-16 [35], before the final redox reaction is carried out by the hydroxylase *Ce*-DAF-9 in partnership with the NADPH-cytochrome P450 reductase *Ce*-EMB-8 [35]. Careful biochemical work originally described DAs as the *Ce*-DAF-12 ligands, the most potent of which is Δ7-DA [25]. More recent work examining metabolites of DAs has described Δ1,7-DA and 3α-OH-Δ7-DA as additional ligands of *Ce*-DAF-12 [36]. When DAs are absent, the co-repressor *Ce*-DIN-1 blocks *Ce*-DAF-12 activity [25,37]. When DAs are present, they bind *Ce*-DAF-12 and reverse *Ce*-DIN-1 repression [25], allowing *Ce*-DAF-12 to simultaneously increase the transcription of *Ce-let-7* microRNA family members that block dauer-formation pathways [38–41] and initiate a reproductive developmental program that promotes the aerobic catabolism of fatty acids for growth [42].

In parasitic nematodes, the mechanisms controlling iL3 arrest and activation as well as homogonic and heterogonic development in *Strongyloides* spp. are less well-understood [43]. The developmental checkpoint regulating homogonic versus heterogonic development in female post-parasitic L1 is regulated by both strain genetics and temperature, with commitment occurring early in L1 development in *S*. *stercoralis* [44] and the closely related *Strongyloides ratti* [45]. In the *S*. *stercoralis* UPD strain used in this study, >95% of larvae develop via the heterogonic route; other isolates range from fully homogonic to fully heterogonic in their development [46,47]. Similarly, frequencies of homogonic and heterogonic development vary among geographical isolates and over the course of infection in *S*. *ratti* [48]. Temperature also regulates the developmental switch between homogonic and heterogonic pathways in *Strongyloides* spp. In *S*. *stercoralis*, temperatures below 34°C signal post-parasitic L1 to take the heterogonic pathway, while temperatures similar to that of the host, 34°C or above, result in development directly to iL3 [44]. Moreover, the two amphidial neurons ASF and ASI regulate the developmental switch between these two routes; when both neurons are inactivated, the vast majority of post-parasitic L1 develop homogonically even at temperatures below 34°C [49]. This is similar to the regulation of the developmental switch in *C*. *elegans* L1 by the analogous ADF and ASI amphidial neurons [50]. However, no specific cellular signal transduction pathway has been implicated in regulating this developmental switch in *Strongyloides* spp.

In *S*. *stercoralis* and closely related parasitic nematodes, including *S*. *ratti* and *Strongyloides papillosus*, progeny of the single generation of free-living male and female adults invariably form developmentally arrested iL3, which are all genetically female—thus leading to a strictly female parasitic generation [6]. However, this post-free-living developmental fate is not shared by all members of the *Strongyloides* genus, as *Strongyloides planiceps* can produce a limited number of free-living generations of males and females [51], and the evolutionarily more distant *Parastrongyloides trichosuri* can produce apparently unlimited generations of free-living males and females [52]. While the formation of *P*. *trichosuri* iL3 is mediated by a constitutively secreted pheromone [53], similar to *C*. *elegans* dauer pheromone [54,55], this does not appear to be the case for *S*. *stercoralis* because iL3 form in the post-free-living generation regardless of population density. However, *S*. *stercoralis* iL3 arrest does require reduced IIS [17]. Interestingly, application of exogenous Δ7-DA to the post-free-living generation of *S*. *stercoralis* and *S*. *papillosus* prevents iL3 arrest, resulting in rhabditiform L3 and L4 in *S*. *stercoralis* [56] and a second generation of fecund free-living females in *S*. *papillosus* [57], thus suggesting that iL3 arrest may be the result, in part, of diminished DAF-12 signaling. However, the duration of exposure to DA necessary to induce these phenotypes has been unknown.

Of the developmental checkpoints, factors regulating iL3 activation in *S*. *stercoralis* and other parasitic nematodes are perhaps the best studied. *S*. *stercoralis* iL3 exhibit positive chemotaxis and thermotaxis towards a variety of molecules indicative of a host [58], including carbon dioxide [59], sodium chloride [60], urocanic acid [61], and host body temperature [62], with many of these responses mediated by amphidial neurons. Upon entering a permissive host, iL3 quickly resume feeding and development, a process that is mediated in part by ASJ amphidial neurons [63]. Resumption of feeding is accompanied by modulation of insulin-like peptide transcripts [19,20], while inhibition of IIS prevents iL3 feeding [18]. Furthermore, increases in cGMP signaling and DA signaling, by exogenous application of these compounds, trigger iL3 feeding [20,56]. However, to our knowledge, it remains unknown whether *S*. *stercoralis*, or any other parasitic nematodes, produces endogenous ligands for DAF-12.

In this study using *S*. *stercoralis*, we demonstrate that DA modulates the post-parasitic switch regulating the decision between reproductive development and iL3 arrest, with increased DAF-12 signaling favoring reproductive development. Furthermore, we demonstrate that in the post-free-living generation, exposure to DA also effects a shift from iL3 arrest, favoring formation of reproductively developing larvae. In the majority of worms, this commitment to reproductive development occurs within 24–48 hours of DA exposure. We also provide the first evidence for endogenous biosynthesis of *Ss*-DAF-12 ligand, as a blockade of iL3 activation by a chemical inhibitor of cytochrome P450s is partially overridden by administration of DA.

## Methods

The UPD strain of *S*. *stercoralis*, originally isolated from naturally infected dogs in 1976, was maintained and cultured as previously described [64,65].

### Ethics statement

*S*. *stercoralis* was maintained in purpose-bred, prednisone-treated mix breed dogs and in purpose-bred Mongolian gerbils according to protocols 802593 and 804883 approved by the University of Pennsylvania Institutional Animal Care and Use Committee (IACUC). All IACUC protocols, as well as routine husbandry care of the animals, were conducted in strict accordance with the *Guide for the Care and Use of Laboratory Animals of the National Institutes of Health*.

### Developmental switching of *S*. *stercoralis* post-parasitic larvae by Δ7-DA

Post-parasitic L1 of *S*. *stercoralis* that were unexposed to environmental cues affecting development were harvested from the intestines of experimentally infected gerbils at necropsy as follows. Tum/Mon strain *Meriones unguiculatus* (Mongolian gerbils) were experimentally infected with 3,000 iL3 and euthanized 21 days later by CO_2_ asphyxiation in accordance with standards established by the American Veterinary Medical Association. The intestines from individual animals were placed in Dulbecco's Modified Eagle's Medium (DMEM), supplemented with 1 mg/ml gentamicin sulfate, with either 1 μM (25S)-Δ7-DA (CAS 949004-12-0) or ethanol carrier (0.1% ethanol) at 37°C. Post-parasitic L1 were picked from the intestinal debris using a stereomicroscope with a 37°C heated stage. Post-parasitic L1 harvested in DMEM with Δ7-DA were transferred to a 35 x 10 mm nematode growth medium (NGM) plate containing 3 ml of agar and spotted with 300 μl of *E*. *coli* OP50 containing 10 μM Δ7-DA, resulting in a final concentration of 1 μM Δ7-DA (0.1% ethanol), and incubated at 37°C. Post-parasitic L1 harvested in DMEM with ethanol carrier were transferred to NGM plates spotted with *E*. *coli* OP50 containing ethanol carrier and incubated at either 37°C or 22°C. The developmental stage of the developing larvae was recorded for all three conditions at both 24 and 48 hours post-plating. Five biological replicates were performed, and the mean percentages of larvae developing to either filariform iL3 or rhabditiform L3-Adult forms, with the standard deviation, were calculated. Proportions of worms in each developmental class as functions of temperature and presence of Δ7-DA were analyzed by 2-way ANOVA with *post hoc* comparisons of selected frequencies by the Bonferroni test.

### Induction of rhabditiform post-free-living L3-L4 forms by Δ7-DA: Assessing dose dependency

Petri dishes (35 x 10 mm) containing 3 ml of NGM agar were seeded with 300 μl of a suspension of *E*. *coli* OP50 in LB broth containing Δ7-DA at concentrations ranging from 331 nM to 10 μM. Assuming uniform dispersal of the compound in the agar, this resulted in NGM/OP50 plates containing 33.1 nM to 1 μM Δ7-DA. Control plates containing 0.1% ethanol, the maximal concentration of Δ7-DA carrier to which the worms were exposed, were made by spotting plates with OP50 suspensions containing 1% ethanol. Semi-synchronous populations of *S*. *stercoralis* eggs were prepared on experimental and control plates by transferring 20–30 gravid free-living *S*. *stercoralis* females to each plate and allowing them to oviposit for three hours at room temperature. At the end of this interval, egg-laying worms were removed from the plates, which then contained cohorts of 50–200 eggs. Plates with eggs were then sealed with Parafilm and incubated for 72 hours at 22°C. Following this incubation, developing worms were classed as rhabditiform L1-L2, filariform iL3, or rhabditiform L3-L4. Mean percentages of worms in each developmental class from three experimental replicates, with standard deviations, were plotted as a function of Δ7-DA concentration. The EC50 for induction of rhabditiform L3-L4 forms was calculated by non-linear regression of frequency in the L3-L4 developmental class on log-transformed Δ7-DA concentrations.

### Kinetics of rhabditiform L3-L4 induction by Δ7-DA: ascertaining a discrete, early triggering event vs. the requirement for continuous exposure

We established triplicate semi-synchronous cultures of post-free-living *S*. *stercoralis* larvae in the same range of Δ7-DA concentrations as described above for the dose-response assessment. At intervals of 24 and 48 hours in culture at 22°C, worms from one of the triplicate cultures at each Δ7-DA concentration were washed off the plate with M9 buffer and then subjected to two additional washes in 10 volumes of M9 buffer; subsequently, the worms were re-plated on non-DA-treated plates and cultured for the balance of the standard 72-hour culture period at 22°C. Worms cultured for 72 hours in the presence of Δ7-DA constituted the continuously exposed controls. At the end of the 72-hour incubation, developing *S*. *stercoralis* larvae were classed as rhabditiform L1-L2, filariform iL3, or rhabditiform L3-L4 as before. Mean percentages, and standard deviations, of worms in the L3-L4 class in each concentration of Δ7-DA were calculated for three experimental replicates. Effects of Δ7-DA exposure duration and concentration were analyzed by 2-way ANOVA with *post hoc* pairwise comparisons of frequencies by the Bonferroni test.

### *S*. *stercoralis* iL3 *in vitro* activation

*In vitro* activation of *S*. *stercoralis* iL3 was performed as previously described [18,20,63] with the following adaptations. All conditions were supplemented with antibiotics (final concentrations: 100 U/ml penicillin, 10 μg/ml streptomycin, and 12.5 μg/ml tetracycline). iL3 were isolated from seven-day-old charcoal coprocultures (incubated at 25°C) by the Baermann technique at 27–29°C. iL3 were subsequently washed twice in deionized water and incubated in M9 buffer [66], supplemented with antibiotics, for three hours at room temperature before distribution amongst the different conditions.

Experiments examining inhibition of iL3 activation with ketoconazole (CAS 65277-42-1; Sigma) were carried out in DMEM supplemented with L-glutamine, 4.5 g/L glucose, and sodium pyruvate (Corning). This medium with the indicated supplements supports resumption of feeding by a majority of *S*. *stercoralis* iL3 at 37°C without additional host-like factors [20]. A 10 mM stock solution of ketoconazole in dimethyl sulfoxide (DMSO) was used for the experimental conditions, which included varying concentrations of ketoconazole (10 μM, 20 μM, 30 μM, 40 μM, and 60 μM) in DMEM, each with 0.8% DMSO. The negative control was M9 buffer, and the positive control was DMEM, each with 0.8% DMSO.

Experiments to ascertain rescue of ketoconazole-mediated inhibition of iL3 activation by Δ7-DA were carried out in DMEM. A 1 mM stock solution of Δ7-DA in ethanol was used for the experimental conditions, which included varying concentrations of Δ7-DA (50 nM, 200 nM, 400 nM, and 800 nM) in DMEM with 35 μM ketoconazole; each condition contained 0.35% DMSO and 0.2% ethanol. The positive controls were DMEM and DMEM supplemented with 800 nM Δ7-DA, while the negative control was M9 buffer; each condition contained 0.35% DMSO and 0.2% ethanol.

Each condition consisted of three wells in a 96-well plate, with approximately 100 iL3 in 100 μl total volume in each well. iL3 were incubated at 37°C in 5% CO_2_ in air for 21 hours; subsequently, 2 μl of fluorescein isothiocyanate (FITC) (CAS 3326-32-7; Sigma) dissolved in N,N-dimethylformamide (DMF) (CAS 68-12-2; Sigma) at 20 mg/ml and incubated for ≥ one month was added to each well, and the cultures were incubated an additional three hours at 37°C and 5% CO_2_ in air (24 hours total). Pre-incubation of FITC solutions was empirically determined to decrease binding of FITC to iL3 cuticles relative to that seen with fresh dilutions of the dye in DMF. iL3 for each condition were pooled and washed five times in 14 ml of M9 buffer, with centrifugation at 75 x g for five minutes at 20°C. iL3 were then mounted on glass slides with grease-edged cover-slips and viewed by fluorescence microscopy using an SZX12 stereomicroscope (Olympus) equipped with an X-Cite 120LED illuminator (Lumen Dynamics). Only live iL3 (indicated by movement) with FITC in the pharynx were scored as "positive" for feeding. Apart from loss of the buccal plug and resumption of feeding, no further development of the iL3 occurs in this system. Dead worms, indicated by lack of movement and/or whole-body FITC staining, were excluded from the feeding analysis, and the percentage of dead worms was determined from the total number of worms present. At least 200 iL3 were counted for each condition. Four biological replicates were performed, and the mean percentage of iL3 feeding in each condition, with the standard deviation, was plotted. Coefficients of correlations between ketoconazole concentration and iL3 feeding and between Δ7-DA concentration and iL3 feeding in the presence of 35 μM ketoconazole were computed by the nonparametric Spearman method. IC50 for ketoconazole inhibition of iL3 feeding and EC50 for Δ7-DA rescue of iL3 feeding in the presence of 35 μM ketoconazole were calculated by non-linear regression of iL3 feeding frequency on concentrations of ketoconazole and Δ7-DA, respectively.

### Identification of *S*. *stercoralis* cytochrome P450s

To identify *S*. *stercoralis* homologs of cytochrome P450-encoding genes, we performed reciprocal BLAST searches of the *S*. *stercoralis* genome v.2.0.4 (available: ftp://ftp.sanger.ac.uk/pub/project/pathogens/HGI/) with *C*. *elegans* cytochrome P450 protein sequences (WormBase release WS245) using Geneious v.6.1.8 (Biomatters Ltd.); BLAST hits were manually annotated using RNAseq data viewed with the Integrative Genomics Viewer v.2.0.34 (available: www.broadinstitute.org/igv/) and Geneious [19,20]. Using mapped and *de novo* assembled RNAseq data, coding sequences were manually corrected to derive full-length coding sequences (S1 Data) and putative protein sequences (S2 Data). Putative cytochrome P450-encoding genes were named, or renamed from those previously described [19], using the standard convention [67]. These genes were identified and related using a combination of reverse-BLAST searches, a protein-identity matrix, and a ClustalW-generated protein alignment (S3 Data) and neighbor-joining phylogenetic tree, with 1000 iterations of boot-strapping, utilizing metazoan cytochrome P450 protein sequences, including *C*. *elegans*, *Caenorhabditis briggsae*, and *Homo sapiens*. All of these analyses were performed with Geneious.

Transcript abundances for each of the *S*. *stercoralis* cytochrome P450-encoding genes were determined as previously described [19,20], with the following adaptations. RNAseq raw reads, derived from polyadenylated RNA libraries, for iL3 (ArrayExpress accession number E-MTAB-2192; http://www.ebi.ac.uk/arrayexpress/experiments/E-MTAB-2192/) [20] as well as *in vivo* activated L3 (L3+), parasitic females, post-parasitic L1, post-parasitic L3 enriched for females, free-living females, and post-free-living L1 (ArrayExpress accession number E-MTAB-1164; http://www.ebi.ac.uk/arrayexpress/experiments/E-MTAB-1164) [19], were mapped to the *S*. *stercoralis* v.2.0.4 genome contigs using Tophat2 v.2.0.13 [68], which utilized Bowtie2 v2.2.4 [69] and Samtools v0.1.18 [70], and previously established parameters [20]. Normalized transcript abundances for cytochrome P450-encoding genes (S4 Data), calculated as fragments per kilobase of coding exon per million fragments mapped (FPKM) with paired-end reads counting as single sampling events, and 95% confidence intervals were determined using Cuffdiff v.2.0.2 [71].

### Data analysis

For all experiments, statistical analyses were carried out and plots were created with Prism version 5.03 (GraphPad Software, Inc.). Statistical probabilities P < 0.05 were considered significant.

## Results

### Exogenous Δ7-DA regulates the developmental fate of post-parasitic larvae of *S*. *stercoralis*

**Fig 2:**
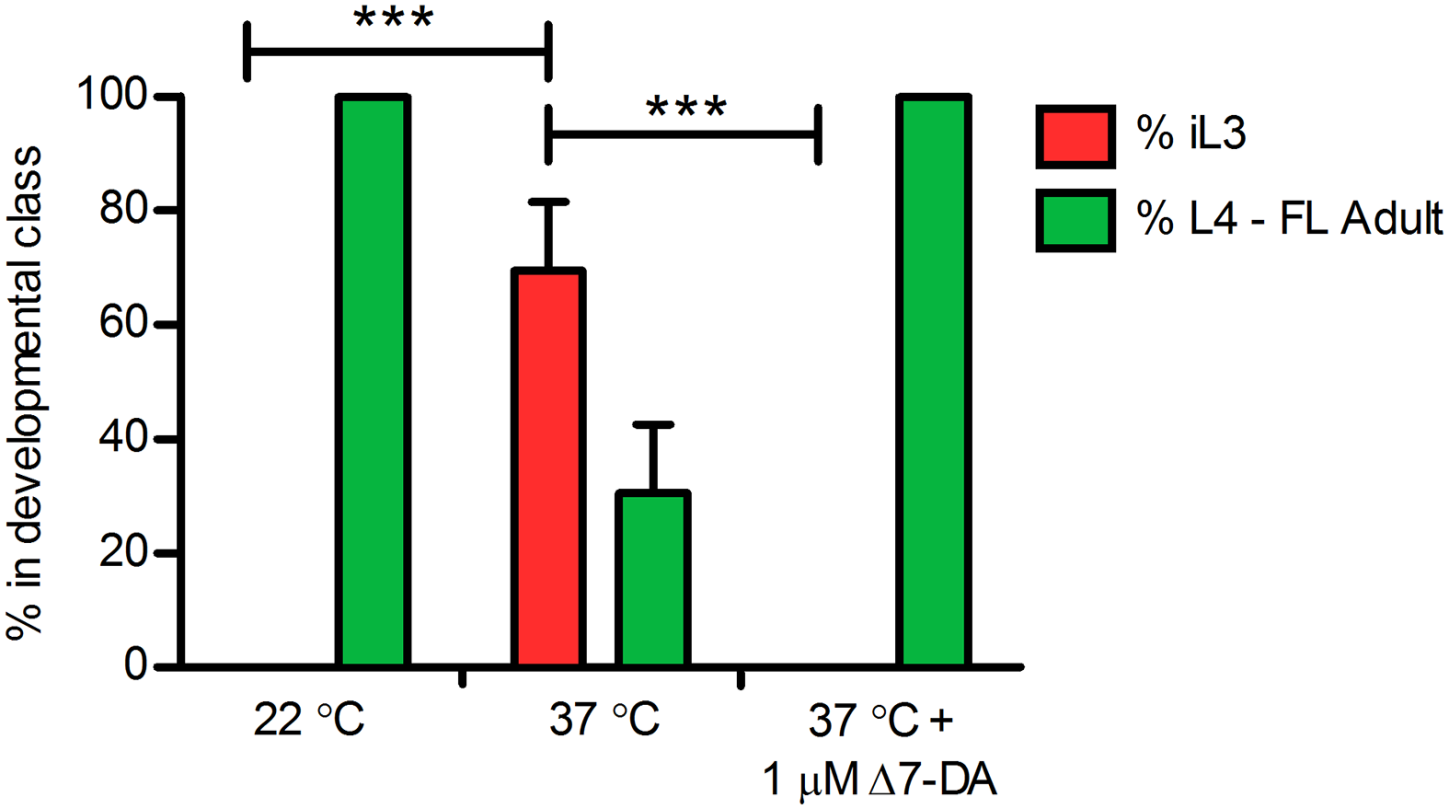
Δ7-Dafachronic acid regulates the developmental switch controlling the homogonic or heterogonic fates of post-parasitic female larvae of *Strongyloides stercoralis*. The frequency distribution of homogonically (red bar) and heterogonically (green bars) developing *S*. *stercoralis* post-parasitic females in cultures maintained at 22°C or at 37°C in the presence or absence of 1 μM Δ7-dafachronic acid (Δ7-DA) was plotted. Data are based on counts of worms, explanted to culture as hatchling first-stage larvae from the intestines of experimentally infected gerbils, scored for development at 24 and 48 hours of culture and assigned to one of two developmental classes representing larvae developing by the homogonic route to infectious third-stage larvae (iL3) or larvae developing via the heterogonic route to free-living females via rhabditiform second, third, and fourth larval stages (L4-FL Adult). Post-parasitic males were excluded from the analysis, as males only develop heterogonically. The bar height represents the mean of five biological replicates and the error bar +1 standard deviation. Brackets indicate statistical comparisons of iL3 frequency at 37°C to frequencies at both 22°C and 37°C with 1 μM Δ7-DA; *** indicates P < 0.001.

Based on its role in promoting continuous reproductive development in *C*. *elegans* [26], we hypothesized that Δ7-DA or a similar *Ss*-DAF-12 ligand promotes heterogonic development by post-parasitic larvae of *S*. *stercoralis* (Fig 1). Conversely, we hypothesized that down-regulation of *Ss*-DAF-12 ligand(s) should drive direct development of post-parasitic L1 to iL3. We tested these hypotheses by administering Δ7-DA to cultures of post-parasitic L1 developing at 37°C, where direct development predominates. As expected, control larvae reared at 22°C developed almost exclusively to free-living adults. However, a mean of 69.4 ± 12.0% (standard deviation) of control larvae reared at 37°C developed directly to iL3, with the remaining minority developing to free-living adults. In contrast to controls reared at 37°C, larvae reared at this temperature in the presence of 1 μM Δ7-DA developed exclusively to free-living adults (Fig 2). These results demonstrate that *Ss*-DAF-12 signaling regulates switching between homogonic and heterogonic developmental alternatives in post-parasitic larvae of *S*. *stercoralis*. Moreover, the ability of Δ7-DA to override the high temperature signal to post-parasitic L1 indicates that *Ss*-DAF-12 signaling operates downstream of mechanisms transducing temperature cues to the larvae from the environment.

### Δ7-DA induces a time-dependent development of free-living fourth-stage larvae among the progeny of free-living males and females

Under normal circumstances, the progeny of free-living male and female *S*. *stercoralis* develop exclusively to iL3 [3]. Just as absence of DA synthesis promotes dauer arrest in *C*. *elegans* [25], we hypothesized that the invariant pattern of iL3 formation that takes place in the post-free-living generation of *S*. *stercoralis* results from absence of *Ss*-DAF-12 ligands (Fig 1). In support, we previously demonstrated that exogenous Δ7-DA can induce a portion of post-free-living *S*. *stercoralis* larvae to bypass iL3 developmental arrest, developing instead to rhabditiform L3-L4 [56]. Moreover, similar concentrations of Δ7-DA are known to induce post-free-living larvae of *S*. *papillosus* to develop to reproductively competent second-generation free-living females [57]. Thus, we sought to achieve a more precise dose-response profile of Δ7-DA induction of development to advanced rhabditiform larvae or adults by post-free living *S*. *stercoralis* larvae; additionally, we sought to ascertain whether this induction occurs in a discrete time interval, or conversely, whether Δ7-DA is required throughout the period of post free-living larval development in order to effect this switch in developmental fate.

**Fig 3:**
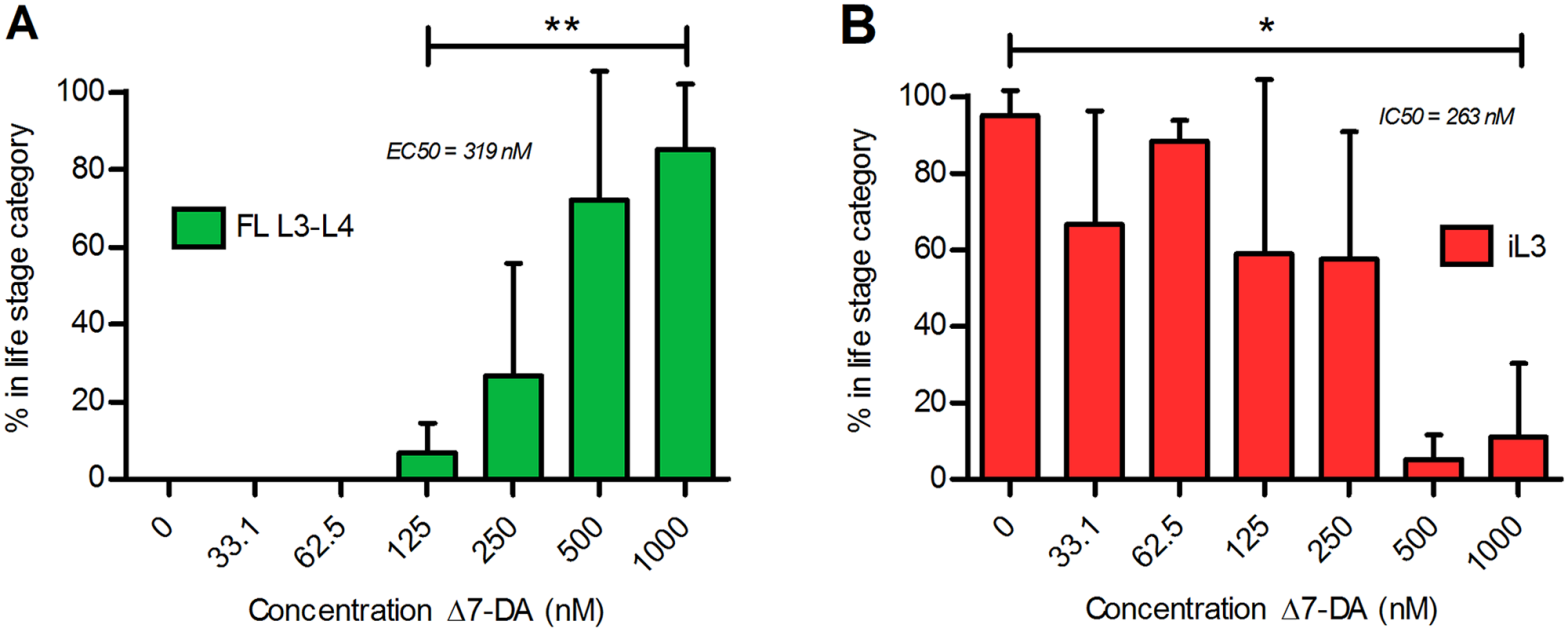
Exogenous Δ7-dafachronic acid promotes formation of *Strongyloides stercoralis* second-generation free-living larvae and blocks the formation of infective third-stage larvae. Post-free-living larvae of *S*. *stercoralis* developing synchronously in agar plate cultures were exposed to increasing concentrations of Δ7-dafachronic acid (Δ7-DA). A) Frequency of development to rhabditiform post-free-living third- and fourth-stage larvae (FL L3-L4) as a function of Δ7-DA concentration. The sample denoted 0 nM Δ7-DA is the ethanol control. ** Positive correlation of FL L3-L4 frequency with Δ7-DA concentration is significant (P = 0.001; R^2^ = 0.902). B) Frequency of development to infective third-stage larvae (iL3) as a function of Δ7-DA concentration, with 0 nM Δ7-DA as the ethanol control. Negative correlation of iL3 frequency with Δ7-DA concentration is significant (P = 0.0135; R^2^ = 0.736). In both panels, bar height represents the mean of three biological replicates; error bars represent +1 standard deviation.

We first cultured semi-synchronous populations of post-free-living *S*. *stercoralis* larvae for 72 hours at 22°C on NGM agar plates with lawns of *E*. *coli* OP50 and fecal bacteria and with Δ7-DA at concentrations ranging from 0 to 1 μM and then assessed the degree of larval development. In the range of 125 nM to 1 μM, Δ7-DA brought about a dose-dependent increase in the frequency of larvae developing to rhabditiform L3 and L4 (Fig 3A). The EC50 for this response was 318 nM, which is comparable to the EC50 of 147 nM for activation of the *Ss*-DAF-12 ligand-binding domain by Δ7-DA in a cell-based reporter assay [56]. As proportions of rhabditiform L3 and L4 increased in response to increasing Δ7-DA concentration, proportions of larvae developing to the iL3 declined (Fig 3B). Proportions of larvae remaining as L1 and L2 remained roughly constant at all concentrations of Δ7-DA (S1 Fig).

**Fig 4:**
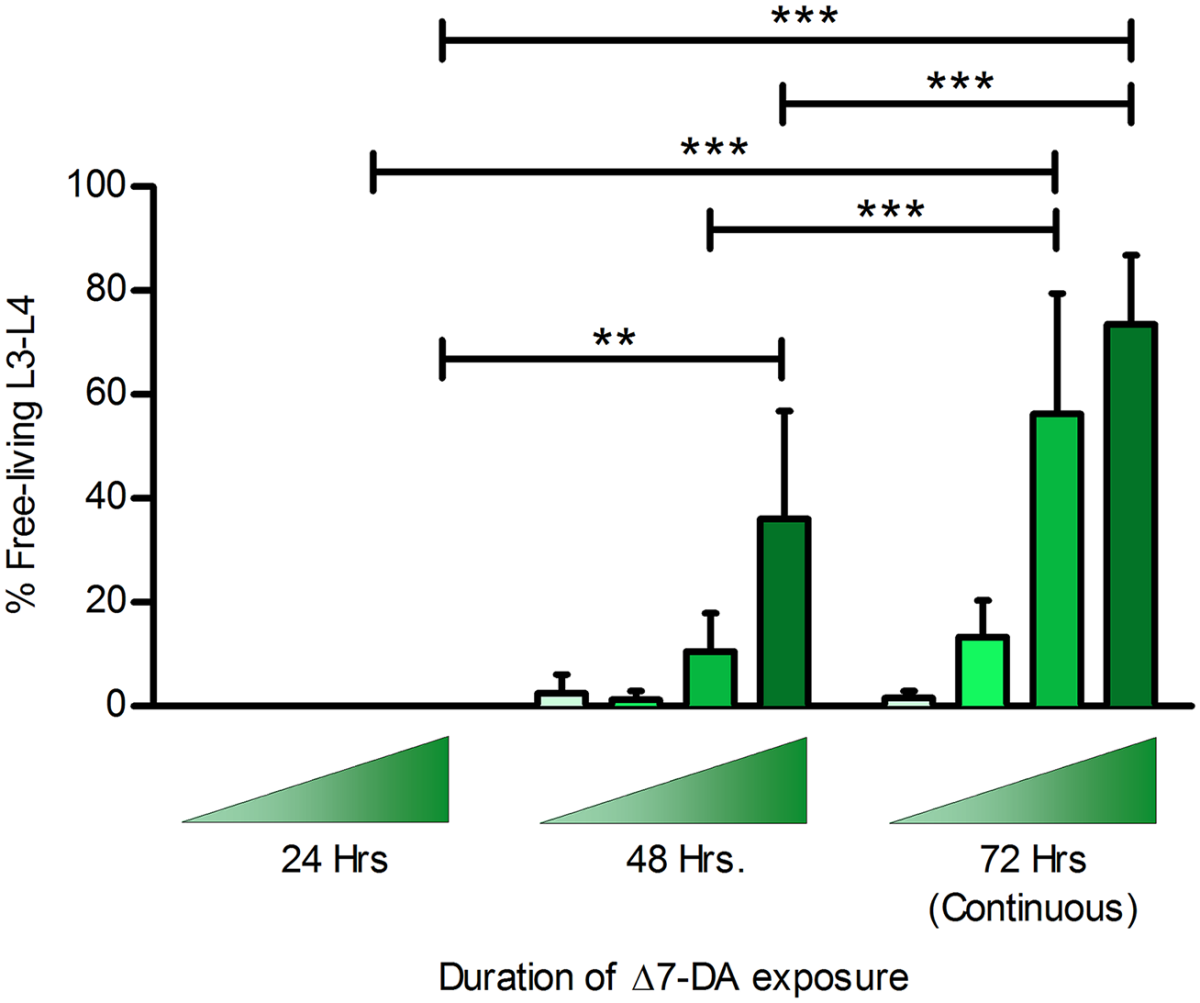
Formation of second-generation free-living larvae in *Strongyloides stercoralis* requires 24–48 hours of exposure to Δ7-dafachronic acid. Frequency of development to second-generation free-living rhabditiform L3 and L4 (FL L3-L4) in *S*. *stercoralis* was plotted as a function of duration of exposure to increasing concentrations of Δ7-dafachronic acid (Δ7-DA). Ascending shaded triangles indicate that developmental frequencies were determined at Δ7-DA concentrations of 125 nM (lightest), 250 nM, 500 nM, and 1,000 nM (darkest). Bar height represents the mean of three biological replicates; error bars represent +1 standard deviation. The overall effects of Δ7-DA exposure duration and concentration were significant, P < 0.0001. For all possible pairwise statistical comparisons of FL L3-L4 frequency with DA exposure durations of 24, 48, and 72 hours, ** P < 0.01; *** P < 0.001.

To ascertain discrete developmental triggering by Δ7-DA, we initiated semi-synchronous plate cultures of post-free-living *S*. *stercoralis* larvae in the presence of increasing concentrations of Δ7-DA. We then washed cohorts of larvae out of the compound at 24 and 48 hours of culture, re-plated them on non-DA-treated plates, and continued culture at 22°C for the balance of the 72-hour culture period. At 72 hours of culture, we compared the frequency of development to rhabditiform L3-L4 in these transiently exposed worms to that of larvae developing for 72 hours at 22°C under continuous exposure to Δ7-DA. None of the larvae exposed to Δ7-DA for the first 24 hours of culture developed to rhabditiform L3-L4 (Fig 4). However, a significant proportion of larvae exposed to Δ7-DA for the first 48 hours developed to rhabditiform L3 and L4. Proportions of larvae undergoing the developmental switch increased with increasing concentration of Δ7-DA. Thus, it appears that between 24 and 48 hours of development at 22°C, a significant proportion of larvae exposed to exogenous Δ7-DA commit to bypassing the iL3 and developing instead to rhabditiform L3-L4. Despite these kinetic refinements, post-free-living *S*. *stercoralis* larvae failed to develop to sexually mature free-living adults, as *S*. *papillosus* larvae do when treated with similar levels of Δ7-DA [57].

### Suppression of feeding by the cytochrome P450 inhibitor ketoconazole in iL3 of *S*. *stercoralis* is reversed by Δ7-DA

**Fig 5:**
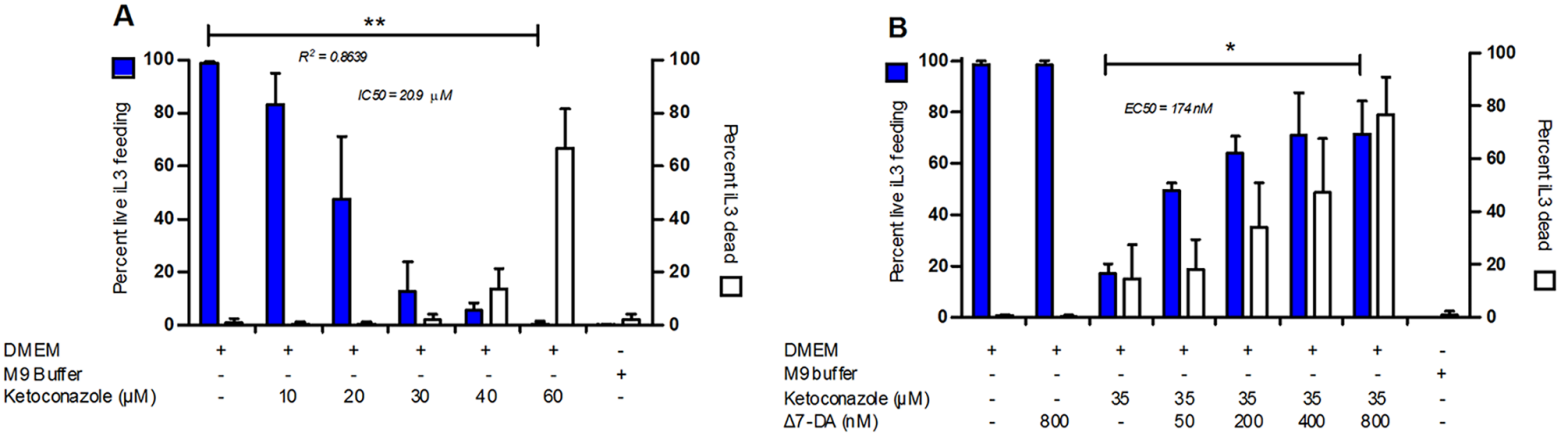
Ketoconazole inhibits developmental activation of *Strongyloides stercoralis* iL3 in host-like culture conditions; this inhibition is rescued by Δ7-dafachronic acid. Frequency of feeding by cultured larvae, as reflected by ingestion of fluorescein isothiocyanate (FITC), was used as an index of developmental activation of infectious third-stage larvae (iL3). Larval mortality was scored as the percentage of larvae classed as non-motile and therefore “dead.” (A) Frequency of iL3 feeding and larval mortality as a function of ketoconazole concentration in Dulbecco’s modified Eagle’s Medium (DMEM), a permissive culture medium. DMEM without ketoconazole was the positive control. M9 buffer, a non-permissive medium, was used without ketoconazole as the negative control. ** Negative correlation of iL3 feeding and ketoconazole concentration (bracketed) is significant (P = 0.0028; Spearman r = -1.000). (B) Frequency of iL3 feeding and larval mortality as a function of Δ7-dafachronic acid (Δ7-DA) concentration in DMEM cultures containing an inhibitory concentration of ketoconazole (35 μM). Cultures of larvae in DMEM alone or in DMEM with 800 nM Δ7-DA were positive controls. Cultures of larvae in M9 buffer constituted the negative control. * Positive correlation of iL3 feeding in 35 μM ketoconazole and Δ7-DA concentration (bracketed) is significant (P = 0.0167; Spearman r = 1.000). In both panels, bar heights represent the mean percentage of iL3 feeding (blue bars) or the mean percentage of dead iL3 (white bars) in four biological replicates. Error bars indicate +1 standard deviation.

If we consider the iL3 of *S*. *stercoralis* to be the developmental equivalent of *C*. *elegans* dauer larvae, then resumption of development by these infectious larvae during the infective process would be the equivalent of dauer recovery [14]. Based on the role of Δ7-DA in promoting continuous development in *C*. *elegans* [25,72], we hypothesized that biosynthesis of this or other *Ss*-DAF-12 ligand(s) is necessary for resumption of development by *S*. *stercoralis* iL3 at the time of infection (Fig 1). In *C*. *elegans*, the cytochrome P450 encoded by *Ce-daf-9* is required for biosynthesis of Δ7-DA from its precursor lathosterone [25,35]. Extending this analogy to *S*. *stercoralis*, we hypothesized that cytochrome P450 activity is required for endogenous biosynthesis of DA(s) or related *Ss*-DAF-12 ligand(s) and that inhibition of this activity would block resumption of development by iL3 at the time of infection. To test this, we asked whether the cytochrome P450 inhibitor ketoconazole could inhibit resumption of development by *S*. *stercoralis* iL3 under host-like *in vitro* culture conditions. Ketoconazole suppressed resumption of feeding by *S*. *stercoralis* iL3 cultured under permissive conditions. The positive control (DMEM) cultures, which reflect host-like biochemical conditions, supported resumption of feeding by a mean of 98.8 ± 0.4% (mean and standard deviation) of the iL3 (Fig 5A). The inhibitory effect of ketoconazole administered to iL3 in DMEM cultures was dose-dependent and maximal at 40 μM and higher, with a mean of 5.6 ± 2.9% of iL3 feeding at 40 μM. In the M9 buffer negative control, 0.1 ± 0.3% of iL3 were feeding.

Ketoconazole broadly inhibits cytochrome P450s [73], and so its suppression of feeding by developing *S*. *stercoralis* iL3 could result from inhibition of multiple such enzymes, including the ortholog of *C*. *elegans* DAF-9. To address this uncertainty, we asked what proportion of the observed suppression of feeding by ketoconazole could be attributable to depletion of Δ7-DA or a similar developmental regulatory steroid from the worms. To this end, we cultured iL3 in permissive medium (DMEM) containing either 35 μM ketoconazole alone or in 35 μM ketoconazole supplemented with increasing concentrations of Δ7-DA. In DMEM with 35 μM ketoconazole, Δ7-DA restored feeding responses among cultured iL3 in a dose-dependent fashion to a maximum of 71.2 ± 16.4% feeding in 400 nM Δ7-DA, compared to 17.1 ± 3.8% feeding in DMEM cultures with 35 μM ketoconazole alone (Fig 5B). This represents a 4-fold increase in feeding over worms cultured in 35 μM ketoconazole alone and accounts for approximately two-thirds of the feeding response seen in non-ketoconazole-treated controls. Mortality, as reflected by the proportion of non-motile (scored as dead) worms, increased as a function of Δ7-DA concentration in the presence of 35 μM ketoconazole (Fig 5B). At concentrations of 200 nM Δ7-DA and higher, the frequency of this mortality was higher than that observed at any concentration of Δ7-DA alone or at comparable levels of ketoconazole (Fig 5A), suggesting a synergistic toxic interaction between ketoconazole and Δ7-DA in this range of concentrations.

### The *S*. *stercoralis* genome contains 26 cytochrome P450-encoding genes

Our findings that the CYP inhibitor ketoconazole suppresses resumption of feeding by iL3 under host-like culture conditions and that this effect can be partially rescued by Δ7-DA suggest that biosynthesis of a steroidal ligand of *Ss*-DAF-12 is necessary for this developmental step. The key enzyme for DA biosynthesis in *C*. *elegans* is the cytochrome P450 DAF-9, which is homologous to the human CYP27A1 that produces bile acids [25]. In order to identify potential homologs of cytochrome P450-encoding genes in *S*. *stercoralis*, including a potential DAF-9 ortholog, we performed reciprocal BLAST searches, followed by manual annotation and correction of the hits to derive putative cytochrome P450 protein sequences (S2 Data). This resulted in the determination of 26 cytochrome P450-encoding genes in *S*. *stercoralis*, which were grouped by family and subfamily by standard cytochrome P450 nomenclature [67]. Of the 26 cytochrome P450s, there were 2 subfamilies with several members which, due to their spatial proximity in the genome and sequence similarity, likely arose as a result of tandem gene duplications. These are the *CYP3A* subfamily, which contains seven genes, and the *CYP29A* family, which consists of nine.

Since *C*. *elegans daf-9* transcripts are up-regulated during reproductive growth and development as well as during dauer exit [28,31,33], we examined the transcript abundance regulation for each of the cytochrome P450 homologs during the *S*. *stercoralis* life cycle (S5 Data and S2 Fig). Congruent with the hypothesis that iL3 activation is mediated by a cytochrome P450, we identified several genes that had an increase in transcript abundance from iL3 to *in vivo* activated L3 –members of the 3A subfamily: *Ss-cyp3a23* and *Ss-cyp3a27*; members of the 29A subfamily: *Ss-cyp29a6*, *Ss-cyp29a13*, *Ss-cyp29a25*, and *Ss-cyp29a26*; *Ss-cyp33e2*; and *Ss-cyp22a9*. We identified *Ss-cyp22a9*, previously identified as *Ss-cyp-9* [19], as the homolog with the closest similarity to *Ce-daf-9*, based on both its phylogenetic relation to *Ce-*DAF-9 (S3 Fig) and its increased transcript abundance in developing larvae and during iL3 activation (S2 Fig). Confirmation of *Ss*-CYP22A9 as the ortholog of *Ce*-DAF-9 awaits future functional studies.

## Discussion

In this study, we hypothesized that DA signaling in *S*. *stercoralis*, through the nuclear hormone receptor DAF-12, would stimulate reproductive growth and development, while decreased DAF-12 activity, resulting from a reduction in DA, would cause developmental arrest. We interrogated several developmental checkpoints in *S*. *stercoralis* to determine whether DA signaling regulates the parasite's developmental program. Our data suggest that DA regulation of DAF-12 signaling plays an important role in the development of iL3 in this pathogen.

While hookworm, filarial worm, and ascarid larvae all constitutively develop to iL3, similar to dauer constitutive (daf-c) mutants in *C*. *elegans* [13], post-parasitic larvae from *Strongyloides* spp. can form a non-obligatory free-living generation of male and female adult worms. However, the molecular mechanisms regulating the developmental switch in the post-parasitic L1 that controls homogonic and heterogonic development have remained elusive. The strain of *S*. *stercoralis* used in this study, the UPD strain, almost exclusively develops via the heterogonic route, whereby post-parasitic female larvae develop to a single free-living generation of adult worms. Other *S*. *stercoralis* isolates have post-parasitic females that develop predominantly via the homogonic route directly to iL3, which are always female, or via a mix of heterogonic and homogonic development [46]. In this study, we demonstrated that developmentally uncommitted female post-parasitic L1 maturing at an elevated temperature, where iL3 arrest normally predominates, may instead be stimulated with exogenous Δ7-DA to develop to free-living adults via the heterogonic pathway (Fig 2). These data not only establish a role for *Ss*-DAF-12 in regulating the post-parasitic L1 checkpoint, but also demonstrate that signaling through this nuclear receptor lies downstream of the pathway sensing thermal cues. This is consistent with findings in *C*. *elegans*, where AFD thermosensitive neurons transduce information through cGMP signaling [74], which lies upstream of DAF-12 signaling [26]. Future studies comparing the effect of DA on post-parasitic L1 development in other *S*. *stercoralis* strains that are genetically predisposed to homogonic development may shed additional light on the requirement for DAF-12 signaling in heterogonic development and the influence of additional signaling pathways on this checkpoint.

*S*. *stercoralis* post-free-living larvae invariably undergo developmental arrest as iL3 in physiological conditions; however, another *Strongyloides* spp., *S*. *planiceps*, can form several successive generations of free-living adults [43,51]. We hypothesized that addition of DA would stimulate *S*. *stercoralis* post-free-living L1 to complete a second free-living generation, similar to that observed with *S*. *papillosus* [57], which also has a single free-living generation in physiological conditions. We found that rearing *S*. *stercoralis* post-free-living larvae in the presence of Δ7-DA suppressed iL3 formation and favored development of rhabditiform L3-L4 (Fig 3), which were morphologically similar to worms undergoing free-living development. While increasing concentrations of Δ7-DA increased the proportion of larvae developing to rhabditiform L3-L4, no free-living adult females were observed. We hypothesize that this difference from the result observed in *S*. *papillosus* may be due to a requirement in *S*. *stercoralis* for additional stimulatory factors or the possibility that Δ7-DA is not the endogenous ligand for *S*. *stercoralis* DAF-12.

We also sought to determine the length of exposure to Δ7-DA required to stimulate *S*. *stercoralis* post-free-living larvae to develop into rhabditiform L3 and L4. Based on work with *C*. *elegans*, where a small quantity of endogenously-produced ligand results in a DAF-12-mediated amplification loop [28], we expected that only a brief pulse of Δ7-DA would be required to prevent iL3 arrest. However, we found that post-free-living larvae required 24–48 hours of exposure to Δ7-DA (Fig 4). This suggests that repressive mechanisms during iL3 arrest actively inhibit DAF-12 function, a phenomenon that could result from: production of DAF-12 antagonists; a decrease in DA precursors by the action of an *S*. *stercoralis strm-1* homolog [75], which is supported by an increase in *Ss-strm-1* transcripts in iL3 [19]; metabolism of DAs into inactive compounds, potentially by other cytochrome P450s that are up-regulated in iL3 (S2 Fig); or the possibility that Δ7-DA is only a weak agonist for *Ss*-DAF-12. We assume that such repressive mechanisms or pharmacokinetic differences are not found in *S*. *papillosus*, where comparable levels of exogenously applied Δ7-DA elicited formation of reproductively competent second-generation free-living females [57].

Δ7-DA causes *S*. *stercoralis* iL3 to resume feeding, which is a hallmark of activation, and initiates a developmental program similar to activation in a permissive host [20,56]. Consequently, we hypothesized that *S*. *stercoralis* endogenously produces DAs or similar *Ss*-DAF-12 ligands during iL3 activation. In *C*. *elegans*, the final biosynthetic step in the production of Δ7-DA is performed by the cytochrome P450 *Ce*-DAF-9, and we hypothesized that a similar enzyme in *S*. *stercoralis* produces endogenous *Ss*-DAF-12 ligands. In order to test this hypothesis, we used ketoconazole, which is a cytochrome P450 CYP3A family inhibitor at nanomolar concentrations and a broad-spectrum P450 inhibitor at micromolar concentrations [73], to broadly inhibit cytochrome P450 function in *S*. *stercoralis* iL3. We found that ketoconazole does indeed inhibit feeding in *S*. *stercoralis* iL3 in a dose-dependent fashion in the micromolar range (Fig 5A), consistent with the synthesis of an endogenous steroid hormone during activation. Inhibition of iL3 feeding by ketoconazole in hookworm species suggests that DA signaling may also be important in iL3 activation in Clade V parasitic nematodes [56,76], which are more closely related to *C*. *elegans* [23]. However, in *S*. *stercoralis*, this ketoconazole-mediated inhibition may be due to a block in the function of a DAF-9 homolog or of one/several of the other cytochrome P450s in the worm. Thus, we sought to determine the extent to which this phenotype could be attributable to the loss of DA by attempting to rescue the ketoconazole-inhibited iL3 by adding back Δ7-DA. We found that approximately two-thirds of the iL3 feeding could be restored by Δ7-DA (Fig 5B), providing evidence that ketoconzaole-mediated iL3 inhibition is due to suppressed production of DA or related *Ss*-DAF-12 ligand(s). Together, these data strongly suggest that *S*. *stercoralis* synthesizes *Ss*-DAF-12 ligands that promote the developmental activation of iL3.

When performing the Δ7-DA rescue experiments, we also noted an increase in iL3 mortality that corresponded with increasing concentrations of Δ7-DA in the presence of ketoconazole (Fig 5B). Since ketoconazole and carrier solution concentrations remained constant, we could only attribute this mortality to an interaction between Δ7-DA and ketoconazole. This synergism suggests ketoconazole may also be blocking the metabolism of exogenous Δ7-DA by inhibition of other cytochrome P450s, allowing toxic levels of the compound or partially metabolized intermediates to accumulate in the worms. This phenomenon might be comparable to the synergism of pyrethrin insecticides when paired with the cytochrome P450 inhibitor piperonyl butoxide [77]. This synergistic effect might be exploited in the potential development of DAF-12 ligands as anthelmintics.

The data we report here support the hypothesis that endogenous production of DA in *S*. *stercoralis* promotes free-living development and iL3 activation, while repression of DAF-12 signaling promotes and maintains iL3 arrest. However, formal proof of this hypothesis awaits discovery and characterization of the natural ligands of *Ss*-DAF-12. Since *S*. *stercoralis* and *C*. *elegans* are phylogenetically distant, as members of separate clades where parasitism is thought to have evolved independently [23], the biosynthesis of steroid hormones may be different in these species. Biochemical- and *in vitro*-based studies, similar to those used to identify Δ7-DA in *C*. *elegans* [25,56], are called for to identify endogenous DAs in *S*. *stercoralis*. Furthermore, additional studies to elucidate the biosynthesis of natural *Ss*-DAF-12 ligands are a significant priority, as this biosynthetic pathway may constitute a chemotherapeutic target in *S*. *stercoralis* and other parasitic nematodes. Our phylogenetic study of the cytochrome P450s in *S*. *stercoralis* (S3 Fig) and of regulation of their transcripts throughout the life cycle (S2 Fig) provide a logical starting point for such biosynthetic experiments. Based upon these findings, we hypothesize that *Ss-cyp22a9* is the functional homolog of *Ce-daf-9* in the parasite. Provided constructs encoding this gene can be optimized for expression in mammalian cells, this hypothesis should be testable using modifications of proven cell-based assay methods [56].

In conclusion, our demonstration that a developmental blockade in *S*. *stercoralis* iL3 by ketoconazole can be rescued by Δ7-DA (Fig 2) constitutes the first functional evidence of DAF-12 signaling stimulated by endogenous synthesis of its ligand in a parasitic nematode. Furthermore, our findings that induction of rhabditiform L3 and L4 in the post-free-living generation by Δ7-DA (Figs 3, 4 and 5) and that homogonic to heterogonic development in post-parasitic L1 is shifted by administration of Δ7-DA (Fig 5) support the hypotheses we frame in this paper about ligation states of *Ss*-DAF-12 and the overall function of *Ss*-DAF-12 signaling during the *S*. *stercoralis* life cycle (Fig 1). The fact that this crucial developmental regulatory signaling pathway can be manipulated by exogenous administration of a steroid ligand, in this case a heterologous one from *C*. *elegans* [25], raises the possibility that *Ss*-DAF-12 signaling may represent a new chemotherapeutic target in *S*. *stercoralis*. This potential for a new class of anthelmintics likely extends to a diverse array of parasitic nematodes, as DAF-12 is conserved in all members of the Strongyloididae investigated to date [57,78] and in hookworms [56,79]. Regarding the latter group of parasitic nematodes, which are phylogenetically diverged from *Strongyloides* spp, the biological activity of the DAs has also been confirmed in *Ancylostoma caninum* [56]. Given the wide range of existing drugs targeting nuclear receptors [80], we propose that the potential of DAF-12 signaling in parasitic nematodes be actively investigated as a novel chemotherapeutic target.

## Supporting Information

S1 Fig Exposure to Δ7-dafachronic acid does not significantly alter the percentage of *Strongyloides stercoralis* post-free-living larvae remaining L1 or L2. *S*. *stercoralis* post-parasitic larvae were hatched out onto plates with concentrations of Δ7-dafachronic acid (Δ7-DA) ranging from 33.1 nM to 1000 nM, as well as an ethanol carrier control. Regardless of Δ7-DA concentration, the percentage of remaining first-stage and second-stage larvae (L1-L2) after 72 hours of culture at 22°C remained roughly the same, with the maximum percentage at 125 nM (34.2 ± 38.3%) and the minimum at 1000 nM (3.7 ± 6.4%). The bar height represents the mean of five biological replicates and the error bar +1 standard deviation.
(TIF)

S2 Fig Transcript abundances of *Strongyloides stercoralis* cytochrome P450-encoding genes are developmentally regulated. *S*. *stercoralis* cytochrome P450 (cyp)-encoding genes were identified in the genome, manually annotated, and named according to the family and subfamily. Both the *Ss-cyp3a* and *Ss-cyp29a* families appeared to have several members resulting from tandem gene duplication events. An *S*. *stercoralis* homolog of *Caenorhabditis elegans daf-9*, *Ss-cyp22a9*, was also identified. Mean transcript abundances, calculated as fragments per kilobase of coding exon per million fragments mapped (FPKM), were determined for the following developmental stages: gravid free-living females (FL Female), post-free-living first-stage larvae (PFL L1), infectious third-stage larvae (iL3), *in vivo* activated third-stage larvae (L3+), gravid parasitic females (P Female), homogonically developing post-parasitic first-stage larvae (PP L1), and homogonically developing post-parasitic approximately third-stage larvae enriched for females (PP L3). Error bars represent 95% confidence intervals.
(TIF)

S3 Fig Putative *Strongyloides stercoralis* cytochrome P450 proteins phylogenetically group with similar metazoan family members. A neighbor-joining phylogenetic tree, with 1000 iterations of boot-strapping, was constructed using cytochrome P450 protein sequences from the following species: *Ascaris suum* (As), *Brugia malayi* (Bm), *Bursaphelenchus xylophilus* (Bx), *Caenorhabditis briggsae* (Cb), *Caenorhabditis elegans* (Ce), *Drosophila melanogaster* (Dm), *Homo sapiens* (Hs), *Hydra vulgaris* (Hv), *Loa loa* (Ll), *Mus musculus* (Mm), *Pristionchus pacificus* (Pp), *Strongylocentrotus purpuratus* (Sp), *Strongyloides ratti* (Sr), *S*. *stercoralis* (Ss), *Takifugu rubripes* (Tr), and *Xenopus laevis* (Xl). The *S*. *stercoralis* CYP22A9 putative protein grouped with *C*. *elegans* DAF-9 and related nematode cytochrome P450s.
(TIF)

S1 Data *Strongyloides stercoralis* cytochrome P450 homolog coding sequences. (FASTA)

S2 Data *Strongyloides stercoralis* cytochrome P450 homolog predicted protein sequences. (FASTA)

S3 Data Protein alignment for cytochrome P450 homologs. (NEX)

S4 Data *Strongyloides stercoralis* cytochrome P450 homolog genome annotations. (GFF3)

S5 Data FPKM values for *Strongyloides stercoralis* cytochrome P450 homologs. (XLSX)
