# Supplementary figures and images for: Regulation of Life Cycle Checkpoints and Developmental Activation of Infective Larvae in Strongyloides stercoralis by Dafachronic Acid
Source: PLoS Pathog. 2016 Jan 4;12(1):e1005358. doi: 10.1371/journal.ppat.1005358 (PMC4703199; doi:10.1371/journal.ppat.1005358)

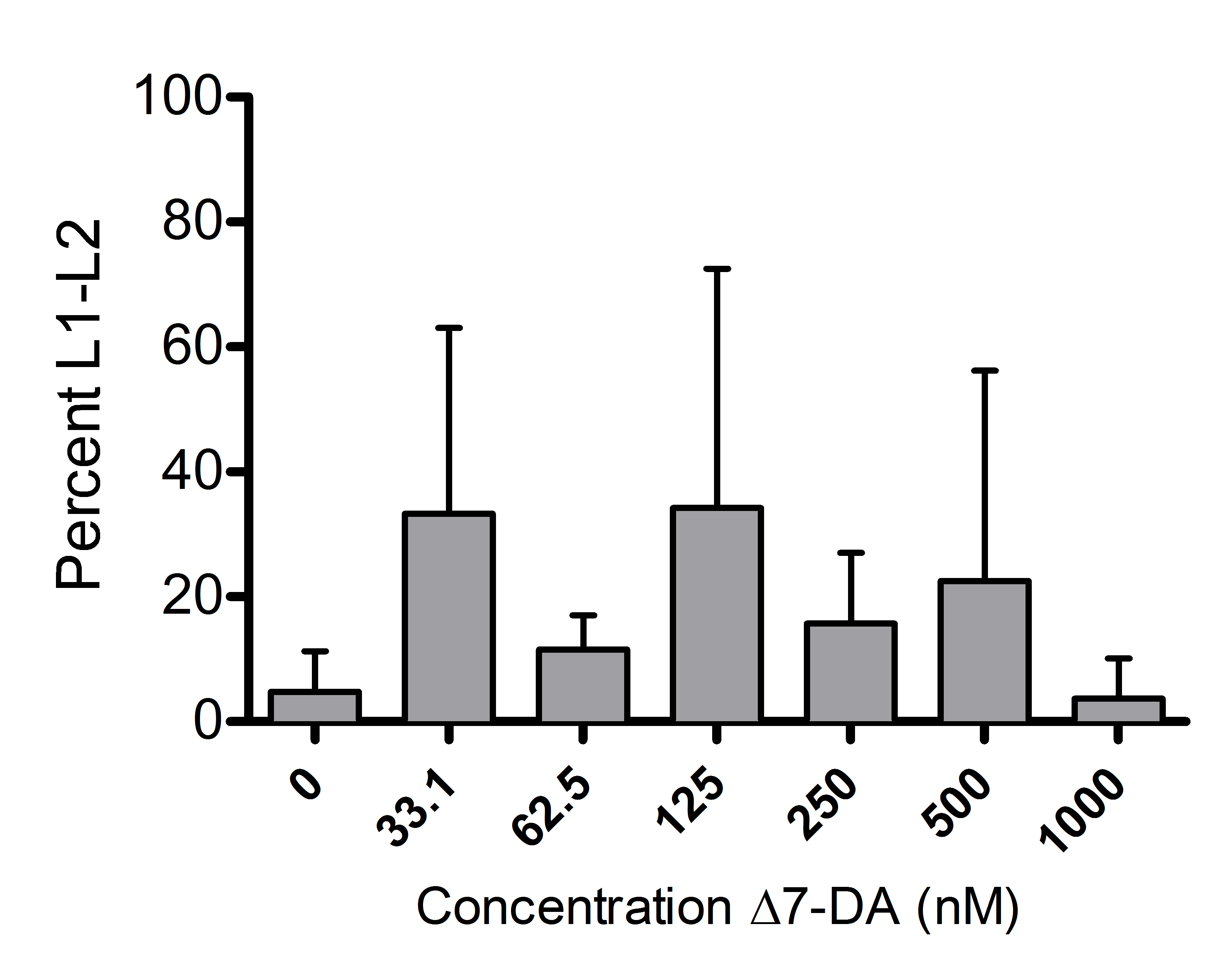

Supplement: S1 Fig — S. stercoralis post-parasitic larvae were hatched out onto plates with concentrations of Δ7-dafachronic acid (Δ7-DA) ranging from 33.1 nM to 1000 nM, as well as an ethanol carrier control. Regardless of Δ7-DA concentration, the percentage of remaining first-stage and second-stage larvae (L1-L2) after 72 hours of culture at 22°C remained roughly the same, with the maximum percentage at 125 nM (34.2 ± 38.3%) and the minimum at 1000 nM (3.7 ± 6.4%). The bar height represents the mean of five biological replicates and the error bar +1 standard deviation. (TIF) [file ppat.1005358.s001.tif]

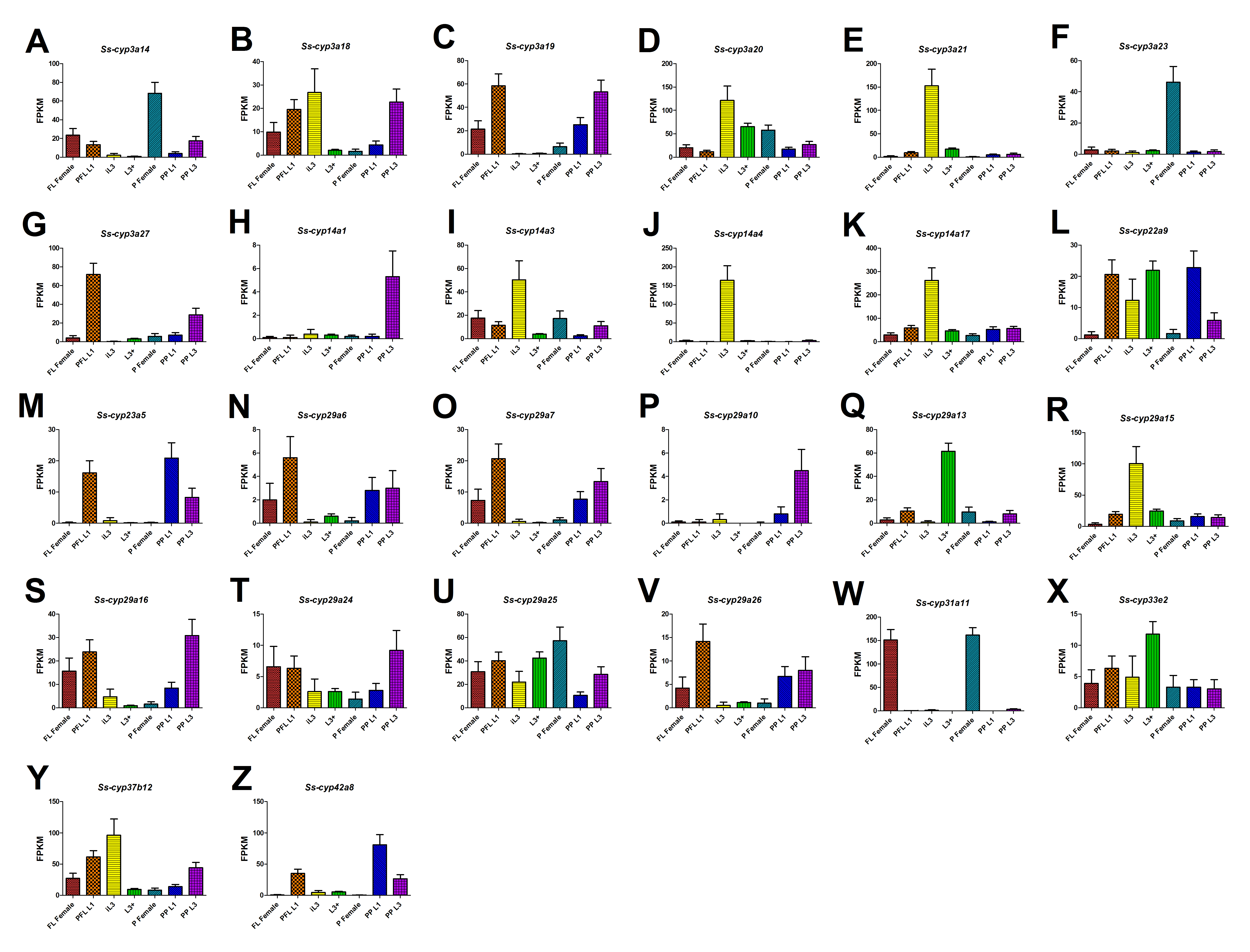

Supplement: S2 Fig — S. stercoralis cytochrome P450 (cyp)-encoding genes were identified in the genome, manually annotated, and named according to the family and subfamily. Both the Ss-cyp3a and Ss-cyp29a families appeared to have several members resulting from tandem gene duplication events. An S. stercoralis homolog of Caenorhabditis elegans daf-9, Ss-cyp22a9, was also identified. Mean transcript abundances, calculated as fragments per kilobase of coding exon per million fragments mapped (FPKM), were determined for the following developmental stages: gravid free-living females (FL Female), post-free-living first-stage larvae (PFL L1), infectious third-stage larvae (iL3), in vivo activated third-stage larvae (L3+), gravid parasitic females (P Female), homogonically developing post-parasitic first-stage larvae (PP L1), and homogonically developing post-parasitic approximately third-stage larvae enriched for females (PP L3). Error bars represent 95% confidence intervals. (TIF) [file ppat.1005358.s002.tif]
